# Antibiotic-Resistant Genes and Bacteria as Evolving Contaminants of Emerging Concerns (e-CEC): Is It Time to Include Evolution in Risk Assessment?

**DOI:** 10.3390/antibiotics10091066

**Published:** 2021-09-03

**Authors:** Alberto Vassallo, Steve Kett, Diane Purchase, Massimiliano Marvasi

**Affiliations:** 1 Department of Biology, University of Florence, 50019 Sesto Fiorentino, Italy; alberto.vassallo@unifi.it; 2 Department of Natural Sciences, Middlesex University London, London NW4 4BT, UK; s.kett@mdx.ac.uk (S.K.); d.purchase@mdx.ac.uk (D.P.)

**Keywords:** ARGs, ARB, antibiotics, antibiotic resistances, risk assessment, evolution

## Abstract

The pressing issue of the abundance of antibiotic resistance genes and resistant bacteria in the environment (ARGs and ARB, respectively) requires procedures for assessing the risk to health. The chemo-centric environmental risk assessment models identify hazard(s) in a dose–response manner, obtaining exposure, toxicity, risk, impact and policy. However, this risk assessment approach based on ARGs/ARB evaluation from a quantitative viewpoint shows high unpredictability because ARGs/ARB cannot be considered as standard hazardous molecules: ARB duplicate and ARGs evolve within a biological host. ARGs/ARB are currently listed as Contaminants of Emerging Concern (CEC). In light of such characteristics, we propose to define ARGs/ARB within a new category of evolving CEC (or e-CEC). ARGs/ARB, like any other evolving determinants (e.g., viruses, bacteria, genes), escape environmental controls. When they do so, just one molecule left remaining at a control point can form the origin of a new dangerous and selection-responsive population. As a consequence, perhaps it is time to acknowledge this trait and to include evolutionary concepts within modern risk assessment of e-CEC. In this perspective we analyze the evolutionary responses most likely to influence risk assessment, and we speculate on the means by which current methods could measure evolution. Further work is required to implement and exploit such experimental procedures in future risk assessment protocols.

## 1. Antibiotic Resistances as Contaminants of Emerging Concern

The development of antimicrobial resistance is a serious public issue that threatens human populations worldwide [1]. Antimicrobial resistances occur in bacteria, viruses, fungi, and parasites that gain the ability to adapt and grow in the presence of antimicrobial compounds, resulting in unmanageable infections [2]. According to the United States Centre for Disease Control and Prevention (CDC), almost 3 million people in the United States become ill because of antibiotic resistance every year, resulting in a minimum of 35,000 deaths [2]. In this context, antibiotic-resistant bacteria (ARB) harboring antibiotic resistance gene(s) (ARGs) can be resistant to antibiotics for several different reasons. They may belong to specific bacterial taxa or possess specific mechanisms of self-protection (e.g., low membrane permeability, presence of efflux pumps, presence of enzymes targeting specific antibiotic classes, modification of the antibiotic target) [3,4,5,6]. Resistance can be included in the chromosome or in a plasmid. In addition to these factors, ARGs can be transferred through the mechanisms of horizontal gene transfer, including transformation, conjugation and transduction, and can be part of mobile genetic elements, such as plasmids and transposons [7,8,9].

There are several factors accelerating the rate of replication and spread of antimicrobial resistances: misuse and overuse of antibiotics, use of antibiotics in agriculture, livestock farming and aquaculture, high income levels, high population density (as in the urban context), easy travel routes and gaps in knowledge [10,11,12,13,14,15,16].

While it is reasonable to expect that high concentrations of antibiotics cause the selection of resistant strains, effects of their minimal concentrations might be overlooked. Even the presence of very low concentrations of antibiotics (i.e., up to several hundred-fold lower than the MIC of susceptible wild-type strains) in the environment can affect ARB selection, as shown by Gullberg et al. [17]. Indeed, it was demonstrated that such limited antibiotic concentrations are sufficient to enrich and maintain either pre-existing or de novo resistant mutants [17]. Co-occurrence of antibiotics and heavy metals is also frequent (e.g., in wastewater treatment plants, soil and water streams near farms, sewage, hospital effluents). More strikingly, in the case of co-occurrence, synergistic effects may cause or even reduce the concentration of antibiotics needed for the selection of resistant bacteria [18]. In vitro tests, including of complex microbial communities using multi-species inocula [19,20] and model biofilms [21,22] to better represent human, animal and environmental microbiomes, confirmed the selective effect of very low antibiotic concentrations. Moreover, more recent experiments employing real wastewater from a hospital and municipal plant to further resemble real environmental conditions, rather than testing antimicrobial agents individually, showed that exposure to hospital effluents positively selected multi-resistant bacteria [23].

Contaminants of Emerging Concern (CEC) are defined as “any anthropogenic or naturally occurring chemicals or microorganisms that are typically not monitored in the environment, but can potentially enter and harm the environment causing adverse ecological and/or human health effects” [24]. CEC include molecules deriving from pharmaceuticals, household products and personal care products. They are detected at low levels in water or the environment with increasing frequency, and there is concern that such compounds may have an impact on life [25]. The list of CEC is still in development, it is expanding [24], and it also includes ARGs and ARB because their increased environmental presence raises concerns for health [26,27]. Krzeminski and colleagues’ review reported comprehensive data concerning full-scale and pilot-scale plants treating real urban wastewater, comparing the CEC removal efficiencies of different technologies. Their CEC list included ARGs and ARB (named “microbial CEC”) [27]. They highlighted the importance of monitoring these two particular kinds of CEC during treatment of wastewater due to their persistence and self-replication potential. However, the ability of ARGs and ARB to evolve was not analyzed. It should be noticed that the presence of bacteria in wastewater treatment plants is routinely monitored; however, these procedures usually aim to check for the presence of specific indicators and do not discriminate between antibiotic-resistant strains and sensitive ones. Moreover, Transformation Products of CEC (TP-CEC), which are compounds derived from CEC and originated through biotic and/or abiotic processes, are also included in CEC lists, and they are getting more attention [24]. They are poorly described in the literature; however, when extending this concept it is reasonable to consider mutated ARGs and ARB similarly to TP-CEC.

**Figure 1:**
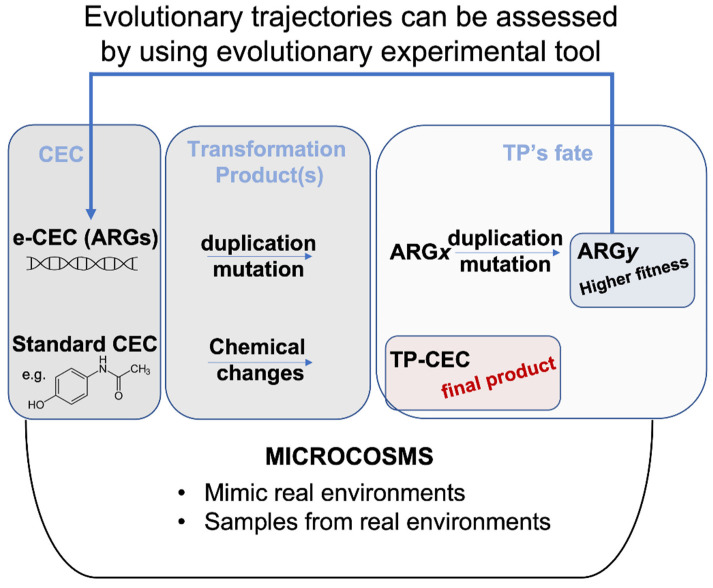
Evolution fate of CEC versus e-CEC. Microcosms are synthetic environments that can help to evaluate the fate of a molecule. Evolutionary trajectories can also be assessed by using evolutionary experiments within microcosms. e-CEC evolve through duplications and mutations, producing many different genes shown in the figure as ARGx, or ARGy. The example in the figure shows that ARGx originates a population (ARGy) with higher fitness. This contrasts with the TP-CEC from a standard molecule (bottom part of the panel) for which the changes terminate with the modification.

Chemo-centric environmental risk assessment involves the identification of hazard(s) in a dose–response manner in animal or in human models [28]. This type of analysis reflects the immediate chemical fingerprint of the tested environment. Therefore, environmental assessment for chemicals relies on models of fate, exposure, toxicity, risk and impacts [28]. Recently, a number of risk assessment procedures have been proposed to cope with the issue of spread of CEC in the environment. For example, different integrated modelling approaches have shown the feasibility of assessing risk for clarithromycin, sulfamethoxazole, diclofenac, ibuprofen, paracetamol, carbamazepine, furosemide, 17-α ethinylestradiol, 17-β estradiol, estrone, perfluorooctanoic acid, perfluorooctane sulfonate, triclosan and polyaniline-based compounds [29,30,31]. All models accommodate temporal and spatial dynamics of environmental conditions (e.g., air, soil and water temperature, water and soil pH, sunlight intensity, etc.), which can substantially affect CEC fate and their recovery in food or soil [29,32]. In standard models of risk assessment, it is also possible to simulate the fate of complex treatments, such as environmental bio-sanitization systems, in which water, for example, is a main conductor. The main difference between ARGs/ARB and standard CEC is that a simple organic CEC (e.g., paracetamol) cannot duplicate itself, nor can it integrate into genomes or mutate; it can only change its molecular structure. Therefore, when high concentrations of standard CEC or related TP-CEC are removed from the environment to establish a new lower concentration, the risk decreases (Figure 1).

The same chemo-centric integrated modelling approaches are less feasible for ARGs and ARB. For example, dose–response curves and exposure assessment data related to ARGs and ARB could help in the immediate risk assessment, but they do not explain how the dose/response will change over time according to evolution [33,34] (Figure 1).

The risk assessment related to ARB is based on detecting the types of microbes in the environment and their metabolic features, discussing exposure, and redacting health studies [35]. Currently, the risk assessment for environmental presence of ARB can be performed by measuring traditional fecal indicator bacteria, such as *Escherichia coli* and enterococci, and possible bacterial pathogens of interest [35]; however, this takes no account of the microbes’ innate capacity to adapt and evolve in response to environmental factors or anthropogenic stimuli.

## 2. Integration of Experimental Evolution in Risk Assessment

ARGs/ARB respond to selective pressure and evolution changing their persistence or replication rate. Changes in temperature, humidity, UV irradiation or environmental contamination can accelerate or lead to drastic deviations in the evolutionary trajectories of ARGs, leading to unpredictable ARB dose/response, rates of ARG horizontal gene transfer and mutation rates. As a mere example, the lytic/lysogenic phase of a phage carrying a resistance gene can be regulated by UV solar irradiation and, depending upon the type of cycle, a new generation of dangerous ARB populations can be generated [36]. Thus, it is clear that understanding of the interactions of many co-varying influences will be required to fully understand the risk. Detection of ARGs’ presence in plasmids, phages and transposons is only one component of the final risk assessment.

Understanding how natural selection can change mutation rates and fitness is crucial for a quantitative description of the microbial evolutionary process. Studies of microbial evolution may bring greater power and precision to risk evaluation. Although some evolutionary experiments may take a long time to complete [37], other trends emerge in short-term experiments. Therefore, here we report rapid and alternative experiments that have been used to assess evolutionary performances of ARGs/ARB in a specific environment and that could be adopted to improve risk assessment protocols. These procedures have the potential to allow monitoring and measurement of stimulus–response relationships and, eventually, modelling.

In general, microcosms can be used to perform such experimental evolutionary studies. Microcosms are simplified ecosystems representing controlled and monitored but otherwise as nearly as possible ‘natural’ environments. Microcosms can be made using water or soil derived from the environment or can be synthesized to mimic a definite environmental aspect. These experiments thus provide a prediction of potential outcomes derived from long-term environmental exposure to ARGs/ARB within a limited experimental time [38]. Moreover, use of multiple microcosms working in parallel also permits the collection of time points that can be sampled and stored in a freezer along an evolutionary trajectory; these can be analyzed later, allowing direct comparisons between ancestral and derived populations [39,40].

Other advantages of such evolution experiments include repeatability [41], and the use of model microbes can facilitate precise control over environmental parameters, population size, mutation and allele selection. However, it should be considered that results obtained with model organisms might not fit real environments, which are surely more complex than in vitro settings.

### 2.1. Antibiotic Bioavailability in the Environment

Recent studies have shown that when antibiotics are detected in soil (even when they are abundant) they are not necessarily bioavailable due to interaction with soil particles and/or other chemicals [42,43,44,45]. Different techniques are available to assess bioavailability of antibiotics, such as the use of gene reporters and the monitoring of gene expression through qPCR or chemical analyses [42,43,44,45]. Another very simple method, called bacterial fitness testing, has been proposed to assess bioavailability of antibiotics in anthropogenically polluted ecosystems [46]. In the fitness tests, pairs of resistant and sensitive bacterial strains are grown, starting from the same number of cells, in a synthetic medium (used as control and in which the assayed antibiotic is assumed to be completely bioavailable and the fitness burden of the resistance cassette is known) and in a microcosm where environmental soil and water are the growth media. The resulting differential fitness of the two strains indirectly reflects antibiotic bioavailability within the two media: if the antibiotic is bioavailable in the microcosm, then it selects the resistant strain; if the antibiotic is not bioavailable in the microcosm, two different outcomes could be observed: (i) the two bacterial populations are equal because of the lack of selective pressure; (ii) populations of the resistant strain decline due to the fitness cost necessary to maintain the resistant determinant [46]. In this context, other methodologies employ specific fluorescent strains to determine how heterogeneous and inhomogeneous conditions influence the selective advantage or disadvantage of antibiotic resistance [39]. The application of such an approach showed that even under the selective pressure of a number of antibiotics, the net selection can be against resistance to the antibiotic in inhomogeneous environments [38].

### 2.2. Hypermutator Strains

Another interesting aspect is that of the hypermutator strains, which have specific genes and/or mutations related to DNA proofreading; these allow ARB to develop a wider spectrum of mutations that can be retained in bacterial populations through environmental selection [47]. This is a further factor to be considered during risk assessment, specifically for ARB. Evaluation of the possibility for ARB to become hypermutators and how mutation rates evolve are thus important parameters to understand many evolutionary processes in hypermutator strains [47]. This can be done by measuring the number of hypermutator key genes (e.g., deficiencies in the DNA mismatch repair system) within the genome or even in a metagenome in the environment within which risk assessment is proposed [48].

### 2.3. Distribution of Fitness Effects (DFE) of Mutations

The concept of fitness is central to microbial evolutionary biology [49]. The fitness effects of ARGs/ARB can bring diverse subpopulations into coexistence, and reversions can quickly change that balance as environmental fluctuations occur. DFE of mutations is a fundamental entity in genetics that describes what proportion of new mutations are advantageous, neutral or deleterious [50]. Advantageous mutations are rare, and the shape of the DFE varies between species and depends on factors such as population size and genome size. For example, in the context of DFE, it was shown that small resistance variability strongly affects evolutionary dynamics for the antibiotic nitrofurantoin, limits resistance development and confines evolution to reproducible mutational paths [51]. The causes of nitrofurantoin’s low resistance variability remain unknown, but in terms of risk assessment this case shows that development of resistance in the tested microcosms was reduced. Thus, development of resistances in real environments may be slower than expected. This is valuable information in terms of risk exposure: specific microcosms will allow systematic investigation of larger sets of antimicrobials, the relation between their empirical evolutionary dynamics and the quantitative determinants of drug resistance evolution [51].

### 2.4. Fitness Tests

Fitness tests are also important to determine the viability of a strain within a population. Generally, mutations providing resistances are costly because they target important biological functions and/or components in the cell; however, this is not always true [52]. In the intestine of pigs, for example, ampicillin-resistant *Escherichia coli* have been shown not to carry a fitness cost for their resistance [53]; similarly, fluoroquinolones’ resistance in *Salmonella enterica* serovar Typhi has been shown not to have a disadvantage over the sensitive parental strain [54]. Both examples suggest drug-resistance mutations can confer antibiotic-independent fitness advantages [55]. This means that removal of the selective pressure may not exert any effect on a particular ARB population.

For the purpose of risk assessment, fitness can be measured with three different methodologies. A first and simplified approach is to study the fitness by calculating the maximum growth rate (*V_max_*) of a culture growing in microcosm. This strategy has been used to study multiple mechanisms of the fitness burden of mupirocin resistance in *Salmonella* Typhimurium [56], nitrofurantoin resistance mechanism and fitness cost in *Escherichia coli* [57], and to study how changes in enzyme function can lead to large fitness effects during adaptive evolution of antibiotic resistance [58].

The second approach, which represents the gold standard for fitness tests in laboratory settings, is based on the generation of microcosms and the use of labelled strains [41]. Strains can be labelled with a fluorescent protein-encoding gene, or simply carry the resistance gene and thus be compared with the control strain lacking the resistance gene. In addition, pairs of mutations in the same resistance genes can be used to compare which mutation is less costly in terms of fitness. With this approach, the strains are incubated in a specific microcosm, replicating the environmental conditions of interest, and then the relative amounts of both genotypes are determined in terms of cells units, CFU/mL, or via cytofluorimetry. The final ratio is generally divided by the initial ratio and converted by using a logarithmic function. More complex calculations can also be generated according to the fitness strategy used, and according to the number of generations that have passed [39]. Lately, other strategies have been proposed to measure fitness. One strategy is based on reducing the fitness differential between the competitors by using a third common reference competitor [39]. Competition experiments in microcosms have been used to study the fitness of antibiotic resistance mutations in *Escherichia coli* in relation to environmental heterogeneity [59]. Another example showed how pollution influences the persistence of the multidrug resistant *Shigella flexneri* 2a YSH6000 strain in polluted river water samples [45]. In these examples the fitness was tested with microcosms using the water from the environment (river water). Yet another example is the measurement of the net selection of model strains for or against resistance in a multi-stress environment [38]. In a multi-stress environment, such as the real environment, aspects of its complexity may favor bacteria for their resistance or sensitivity to a particular antibiotic. Sublethal antibiotic concentration may also induce, or not, a specific resistance. A simple model of the growth of sensitive and resistant strains has been proposed by Chait and collaborators [38]. In this system a YFP (yellow fluorescent protein)-labelled tetracycline-sensitive and a CFP (cyan fluorescent protein)-labelled tetracycline-resistant *E. coli* are mixed and grown together over a diffusing toxin gradient in agar, in a complex environment either with or without a uniform concentration of a specific antibiotic [38]. This type of fitness experiment could provide rapid insight regarding a specific question, for example, if a sublethal antibiotic concentration still exerts selective pressure in a specific microcosm.

Finally, another technical approach is the quantification and comparison of the Minimum Inhibitory Concentration (MIC) against a selected compound [60,61]. In this scenario the cultures are serially challenged in sublethal or lethal doses of antibiotics. Experiments can be done in microcosms (reflecting the real environment) to assess the fate of MIC changes or in defined media. In addition, MIC experiments are low-cost and provide data to be easily compared with EUCAST clinical breakpoints.

### 2.5. Evolution Potential of Mobile Elements and Plasmids

Plasmids and mobile genetic elements are crucial for the spread of ARGs. In the case of ARG-carrying plasmids, their potential ability to promote bacterial adaptation and evolution can be assessed by using plasmid fitness experiments. The fitness effects of plasmids are pivotal because of their ability to undergo horizontal gene transfer, mainly through conjugation, between bacterial hosts and to accelerate the evolution of plasmid-mediated antibiotic resistance [62]. Other mobile genetic elements, such as transposons and integrons, also play a central role in facilitating horizontal genetic exchange and therefore promote the acquisition and spread of resistance genes [63]. As defined by Partridge and colleagues [63], the insertion sequences (IS) and transposons (Tn) are “discrete DNA segments that are able to move themselves (and associated resistance genes) almost randomly to new locations in the same or different DNA molecules within a single cell”. It is clear that for the scope of risk assessment the identification of IS and Tn associated with ARGs could be very helpful in evaluating the ability of a certain ARG to spread in the environment. In this context the use of untargeted metagenome sequencing can provide an approach to identify the presence of specific IS or Tn. To this purpose, several bioinformatic tools are available to predict and identify mobile elements from DNA sequences, such as ISfinder [64], Transposon Registry [65], INTEGRALL [66], and SCC*mec* elements [67]. An interesting future upgrade would be the incorporation of these tools into mathematical modelling [68], providing indicators regarding risk associated with presence of a specific mobile element. It is reasonable to assume that a higher presence of IS or Tn would increase the risk of spreading ARGs, although ARGs could not be necessarily associated to mobile elements.

### 2.6. Evolving Resistance to New Antimicrobials

An experimental prediction of the natural evolution of antibiotic resistance was proposed by Barlow and Hall in 2003 [69]. The experimental setup is reasonably easy and it could be performed in other similar contexts by using microcosms and MIC tests. These authors assessed whether the TEM beta-lactamases, which confer resistance against ampicillin, have the potential to evolve and provide cefepime resistance. The principle is to use an *E. coli* strain grown in increasing concentrations of the new antibiotic (cefepime in this case). *E. coli* was passaged twice through twofold serial dilutions of cefepime, once through ampicillin and then once again through a dilution series of cefepime. Cells collected from culture with the highest tolerated concentration of cefepime were used to inoculate the following culture and, after multiple passages in presence of cefepime, eight independent populations reached a MIC equivalent to that of clinical isolates [69]. Measurements of how fast this adaptation to new antibiotics occurs might be implemented in risk assessment.

## 3. Conceptual Framework

“Complexity”, “decision-making”, “environmental risk assessment”, “modelling”, “regulations”, “toxicity” and “validation” are all important keywords related to the complex matter of the fate and evolution of an e-CEC in the environment. We are far from being able to integrate or unravel the resolution of just one of these words in risk assessment. Nevertheless, mathematical modelling and metadata analyses are the tools that can integrate and implement the evolutionary data into a predictive risk algorithm. A recent review by Knight and collaborators [68] discusses the opportunity to predict antibiotic resistance evolution with mathematical models and to generate the evidence base needed to help policies in managing antibiotic resistance. It is clear that in this context the application of such empirically observed evolutionary experiments to support mathematical modelling could provide a solid foundation for ARB/ARG control policy and risk assessment. Another approach is to include data-mining for metadata analysis and adopt the screening of literature to define a behavior or a trend; for example, the fitness costs associated with single mutational events that confer resistance have been determined via metadata analysis [52]. This approach has unraveled a simple fact: the strategy of stopping the use of a drug once resistance has evolved is not always effective in eliminating resistance.

**Figure 2:**
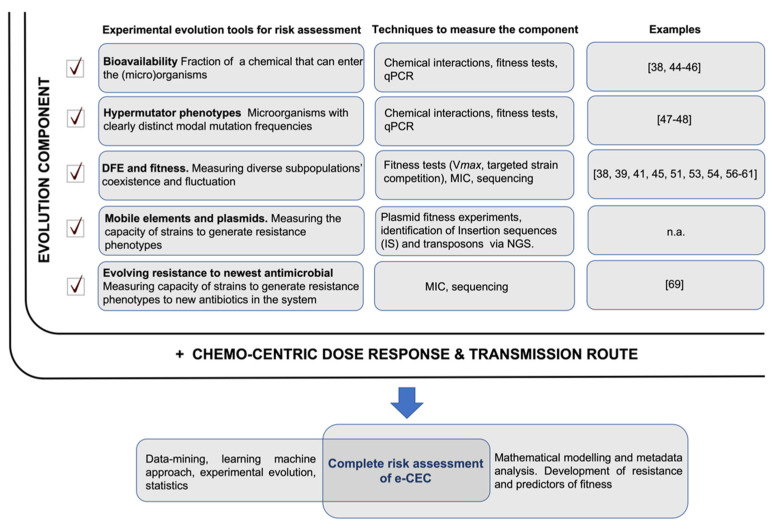
ARGs/ARB risk assessment. The full risk assessment would include two components: the ‘evolution component’, which is the sum of different evolutionary trajectories measurable in microcosms or directly in the environment (highlighted with a checkmark), and ‘the chemo-centric dose–response and transmission route’ risk. The evolutionary trajectories measure the evolution of ARGs/ARB (their probable future) and their likely persistence in the system. This complements a dose–response risk assessment that analyses the immediate condition but takes no account of future state changes of ARGs/ARB. Relevant papers showing possible practical uses of the experimental evolution tools are reported; n.a.—not available.

A model of risk assessment integrating experimental evolution tools is shown in Figure 2.

The complete risk evaluation of ARGs/ARB should consider both the “evolution component” and the “dose–response factor” (Figure 2). Dose–response and transmission route assessments would reveal the immediate picture of the risk related to the presence of ARGs/ARB. The evolutionary part will propose an assessment on a long-term trajectory along with its potential evolutionary paths and fluctuations due to environmental changes. References showing possible applications of experimental evolution tools are listed in Figure 2; it must be specified that these examples are models, and they need adaptation to best fit real environmental conditions. Not all the proposed evolutionary tools in Figure 2 could reasonably be integrated, but any addition of such experimental evidence will better identify the evolutionary trajectory of ARGs/ARB in real environments. As understanding grows, new algorithms can be developed and integrated to better assess the contribution of all factors to overall risk.

**Figure 3:**
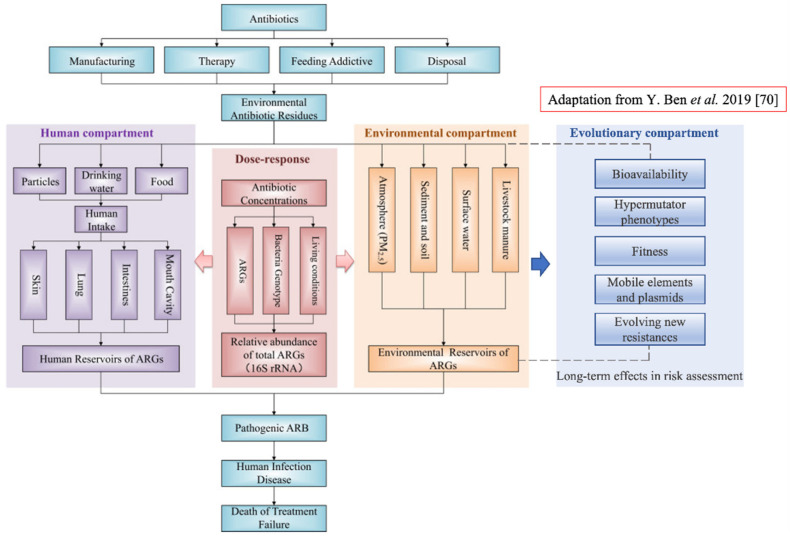
Conceptual framework of human health risk assessments of antibiotic resistance and the new component ‘Evolutionary compartment’ (on the right in blue). The evolutionary component has link-bridges with the ‘Environmental component’, and by extension it affects the ‘Human compartment’ leading to the fate of the entire system. The ‘Evolutionary component’ would introduce a specific portion of assessment regarding microorganisms’ evolution within a specific environment; this will provide assessment of long-term effects in ARB and ARGs selection and persistence (Figure revised from [70]).

How to practically introduce these concepts in risk assessment? One of the overall goals of health risk assessment, as suggested in [70], would be to measure exposure to antibiotic resistance and to estimate the number of infectious diseases caused by specific ARBs isolated from a specific environment. To this end, the implementation of an evolutionary test of fitness on the same ARBs would allow an estimation of potential future trajectories. This would help to assess whether or not the number of infectious diseases is likely to increase in the future. A conceptual framework of human health risk assessments of antibiotic resistance including the new component is proposed in Figure 3.

## 4. Conclusions

As microorganisms evolve in a specific environment, this will provide long-term effects in ARB and ARGs selection and persistence. In this work, we propose to define ARGs/ARB within the new category of evolving CEC (or e-CEC). When they escape in the environment, these e-CEC could constitute the origin of a new dangerous and selection-responsive population. Consequently, it is time to acknowledge this trait, to include evolutionary concepts within modern risk assessment of e-CEC and to implement it with evolutionary tests on fitness of ARB.
